# Neural correlates of digital measures shown by structural MRI: a post-hoc analysis of a smartphone-based remote assessment feasibility study in multiple sclerosis

**DOI:** 10.1007/s00415-022-11494-0

**Published:** 2022-12-05

**Authors:** Marco Ganzetti, Jennifer S. Graves, Sven P. Holm, Frank Dondelinger, Luciana Midaglia, Laura Gaetano, Licinio Craveiro, Florian Lipsmeier, Corrado Bernasconi, Xavier Montalban, Stephen L. Hauser, Michael Lindemann

**Affiliations:** 1 F. Hoffmann-La Roche Ltd, Basel, Switzerland; 2 Department of Neurosciences, University of California San Diego, San Diego, CA USA; 3 Department of Neurology-Neuroimmunology, Centre d’Esclerosi Múltiple de Catalunya (Cemcat), Hospital Universitari Vall d’Hebron, Barcelona, Spain; 4 Department of Medicine, Autonomous University of Barcelona, Barcelona, Spain; 5 Department of Neurology, University of California San Francisco, San Francisco, CA USA; 6 Present Address: Novartis Institutes for Biomedical Research, Basel, Switzerland

**Keywords:** Multiple sclerosis, Smartphone sensor, MRI, Neural correlates, Digital health technology tools

## Abstract

**Background:**

A study was undertaken to evaluate remote monitoring via smartphone sensor-based tests in people with multiple sclerosis (PwMS). This analysis aimed to explore regional neural correlates of digital measures derived from these tests.

**Methods:**

In a 24-week, non-randomized, interventional, feasibility study (NCT02952911), sensor-based tests on the Floodlight Proof-of-Concept app were used to assess cognition (smartphone-based electronic Symbol Digit Modalities Test), upper extremity function (Draw a Shape Test, Pinching Test), and gait and balance (Static Balance Test, Two-Minute Walk Test, U-Turn Test). In this post-hoc analysis, digital measures and standard clinical measures (e.g., Nine-Hole Peg Test [9HPT]) were correlated against regional structural magnetic resonance imaging outcomes. Seventy-six PwMS aged 18–55 years with an Expanded Disability Status Scale score of 0.0–5.5 were enrolled from two different sites (USA and Spain). Sixty-two PwMS were included in this analysis.

**Results:**

Worse performance on digital and clinical measures was associated with smaller regional brain volumes and larger ventricular volumes. Whereas digital and clinical measures had many neural correlates in common (e.g., putamen, globus pallidus, caudate nucleus, lateral occipital cortex), some were observed only for digital measures. For example, Draw a Shape Test and Pinching Test measures, but not 9HPT score, correlated with volume of the hippocampus (*r* = 0.37 [drawing accuracy over time on the Draw a Shape Test]/ − 0.45 [touching asynchrony on the Pinching Test]), thalamus (*r* = 0.38/ − 0.41), and pons (*r* = 0.35/ − 0.35).

**Conclusions:**

Multiple neural correlates were identified for the digital measures in a cohort of people with early MS. Digital measures showed associations with brain regions that clinical measures were unable to demonstrate, thus providing potential novel information on functional ability compared with standard clinical assessments.

**Supplementary Information:**

The online version contains supplementary material available at 10.1007/s00415-022-11494-0.

## Introduction

Multiple sclerosis (MS) is a chronic disease of the central nervous system [1]. A long-held view of MS is of a multi-focal, immune-mediated, inflammatory-demyelinating, and degenerative disorder of the white matter. Recent structural magnetic resonance imaging (MRI) studies, however, have revived the important role of cortical pathology in the pathophysiology of MS and in the rate of disability progression [2].

MRI-measured atrophy occurs in distinct, non-random patterns and involves many different regions, including the cerebral cortex, deep gray matter, brainstem, cerebellum, and spinal cord [2–7]. The rate of atrophy differs from region to region [6]. However, the progression of regional atrophy tends to follow specific sequences depending on the MS phenotype. In relapse-onset MS, the first regions affected by atrophy include the posterior cingulate cortex and precuneus, while the globus pallidus and medial precentral gyrus are among the last regions to become atrophic. In primary progressive MS, by comparison, the first regions to become atrophic are the thalamus, cuneus, and precuneus, and the last regions to show signs of atrophy are the frontal operculum and middle temporal gyrus [7].

Although there have been efforts to link clinical disability to regional atrophy [8–12], the relationship between digital measures of functional ability and MRI outcomes has not been extensively studied. Previously we showed that digital measures obtained with the Floodlight Proof-of-Concept (PoC) app in people with MS (PwMS) correlate with global MRI outcomes, in particular with total brain volume [13]. However, this analysis did not consider the regional specificity of cortical, deep gray matter, infratentorial, and spinal cord pathology. Here we extend this prior work by assessing the regional neural correlates of an expanded set of digital measures.

## Methods

### Study design and participants

This 24-week, non-randomized, interventional, feasibility study (clinicaltrials.gov: NCT02952911) aimed to assess the feasibility of remotely monitoring PwMS with the Floodlight PoC app, through sensor-based assessment via a provisioned smartphone device [14]. The study design, inclusion, and exclusion criteria have been previously reported [13, 14]. The study enrolled 76 PwMS and 25 healthy controls aged 18–55 years, from two separate sites in the USA (University of California San Francisco, San Francisco, CA) and Spain (Multiple Sclerosis Centre of Catalonia, Vall d’Hebron University Hospital, Barcelona); MRI data were only collected from PwMS. PwMS were diagnosed according to the 2010 revised McDonald criteria [15] and had an Expanded Disability Status Scale (EDSS) [16] at baseline of 0.0–5.5. A sample size of least 70 PwMS was considered adequate to detect a linear correlation coefficient of 0.33 with > 80% power.

### Floodlight PoC app

**Fig. 1:**
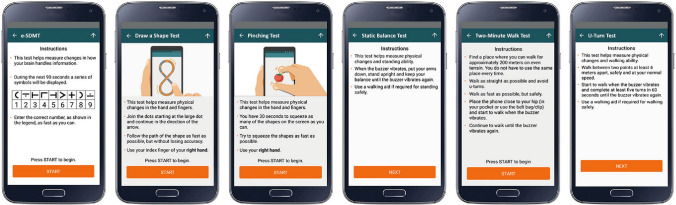
Screenshots of the smartphone sensor-based tests included in the Floodlight PoC app. Screenshots depict the instruction screens for the smartphone-based electronic Symbol Digit Modalities Test, Draw a Shape Test, Pinching Test, Static Balance Test, Two-Minute Walk Test, and U-Turn Test. *e-SDMT* smartphone-based electronic Symbol Digit Modalities Test*, PoC* Proof-of-Concept

At baseline, all participants received a pre-configured smartphone (Samsung Galaxy S7) with the Floodlight PoC app pre-installed [13, 14]. The app prompted all participants to perform daily and weekly sensor-based tests of functional ability (referred to as “active tests”) at home without supervision by a physician or nurse, in three key domains affected by MS. Cognition was assessed by the smartphone-based electronic Symbol Digit Modalities Test (e-SDMT), upper extremity function by the Draw a Shape Test and Pinching Test, and gait and balance by the Static Balance Test (SBT), Two-Minute Walk Test (2MWT), and U-Turn Test (UTT) (Fig. 1). Performance on each active test was quantified by a set of digital measures extracted from the sensor signals that are illustrative of the test (Table 1).

**Table 1:** Outcome variables

Floodlight PoC app				
Functional domain	Smartphone sensor-based active test	Functional subdomain	Digital measure^a^	Higher scores indicate performance that is:
Upper extremity function	Draw a Shape Test	Accuracy	Mean trace accuracy	Better
		Swiftness of movement	Mean trace celerity, 1/s	Better
		Smoothness of movement	CV of linear, angular, and radial drawing velocity	Worse
	Pinching Test	Accuracy	Total number of successful pinches, *n*	Better
		Responsiveness	Gap time between pinch attempts, s	Worse
		Pinching asynchrony	Double touch asynchrony of successful pinches, s	Worse
Cognition	e-SDMT	Information processing speed	Number of correct responses, *n*	Better
			Maximum gap time between correct responses, s	Worse
		Cognitive fatigue	Speed fatigability index of the last 30 s	Better
Gait and balance	SBT	Postural control	Sway path, m/s^2^	Worse
	UTT	Turning ability (dynamic balance and gait)	Turn speed, rad/s	Better
	2MWT	Gait pace	Step frequency, Hz	Better
		Gait variability	Step frequency variance, Hz^2^	Worse
		Gait intensity	Step power, m^2^/s^3^	Better

Normalized volumes derived from brain MRI scans, mL^b^			
Cerebral cortex gray matter	Deep gray matter	Brainstem and upper cervical cord area	Other
1. Middle frontal 2. Superior frontal 3. Lateral and medial orbitofrontal 4. Precentral 5. Postcentral 6. Paracentral 7. Opercularis, triangularis, orbitalis 8. Insula 9. Precuneus 10. Cuneus, pericalcarine, lingual 11. Anterior cingulate 12. Posterior cingulate 13. Isthmus cingulate 14. Parahippocampus, fusiform, entorhinal 15. Superior parietal 16. Supramarginal 17. Inferior parietal 18. Lateral occipital 19. Inferior and middle temporal 20. Superior and transverse temporal	1. Thalamus 2. Hippocampus 3. Putamen 4. Globus pallidus 5. Caudate nucleus 6. Amygdala 7. Accumbens	1. Midbrain 2. Pons 3. Medulla oblongata 4. Upper cervical cord area^c^	1. Lateral ventricles 2. Cerebral white matter 3. Corpus callosum area 4. Cerebellum—hemispheres 5. Cerebellum—vermis

*2MWT* Two-Minute Walk Test, *e-SDMT* smartphone-based electronic Symbol Digit Modalities Test, *PoC* Proof-of-Concept, *SBT* Static Balance Test, *UTT* U-Turn Test

^a^Computational definitions are provided in Table SI1

^b^Volumes were calculated for all regions with the exception of the corpus callosum and the upper cervical cord for which areas measured in mm^2^ were calculated

^c^Upper cervical cord area was determined as mean area along C2–C3 levels

#### Smartphone sensor-based tests

Key neurologic functions underlying cognitive information processing speed were assessed by the e-SDMT. It instructed participants to correctly match as many symbols as possible to their paired digits within 90 s, according to a symbol–digit key also displayed on the smartphone screen.

The Draw a Shape Test assessed fine finger movements, or dexterity; participants were prompted to draw six pre-written shapes of increasing complexity (two diagonal lines, a square, a circle, a figure-of-8, and a spiral). By comparison, the Pinching Test measured fine pinching or grasping dexterity. Participants were instructed to successfully pinch as many circular tomatoes appearing on the screen as possible within 30 s, with new tomatoes appearing at random locations.

Finally, gait and balance were assessed by three active tests: the SBT, 2MWT, and UTT. The SBT required participants to stand as still as possible for 30 s. The 2MWT assessed regular, straight walking for 2 min. The UTT, which measured both dynamic balance and gait, instructed participants to perform five consecutive U-turns that were at least 4 m apart, within a minute. The use of an assistive device and/or orthotic was permitted as needed while performing the 2MWT and UTT.

#### Digital data processing

Since the active tests were unsupervised, quality control flags as defined in Montalban et al. [13] were applied to identify tests that were not performed in accordance with the test’s instructions and to ensure accurate interpretation of the collected data. This included flags to identify attempts of “play to quit” (i.e., trying to complete an active test as quickly as possible without performing the instructed tasks) or instances where the smartphone device was kept on a table during the gait and balance tests. Any test identified by one of these flags was excluded from the analyses. All remaining tests were considered as valid tests. Only participants who contributed on average at least 1.5 valid tests per week (corresponding to 21% adherence) were included in the analysis.

### MRI acquisition and processing

#### Image acquisition

Brain MRI scans were collected from PwMS at baseline and at week 24 with Siemens 3-Tesla scanners (Siemens Healthcare GmbH, Erlangen, Germany) following optimized clinical practice protocols in place at the two investigating centers: (1) Hospital Universitari Vall d'Hebron, three-dimensional (3D) T1-weighted (repetition time [TR] = 2300 ms; echo time [TE] = 2.98 ms; inversion time [TI] = 900 ms; voxel size = 1 × 1 × 1 mm) fluid-attenuated inversion recovery (FLAIR; TR = 6000 ms; TE = 394 ms; TI = 2100 ms; voxel size = 1 × 1 × 1 mm) sequences acquired in a Siemens Trio Tim; (2) University of California San Francisco, 3D T1-weighted (TR = 2300 ms; TE = 2.98 ms; TI = 900 ms; voxel size = 1 × 1 × 1 mm) FLAIR (TR = 5000 ms; TE = 389 ms; TI = 1,800 ms; voxel size = 1 × 1 × 1 mm) sequences acquired in a Siemens Skyra.

#### Image processing

MR images were analyzed with icobrain ms v.5.0 (icometrix, Leuven, Belgium), a CE- and Food and Drugs Administration-certified software as medical device for automatic labeling, visualization, and volumetric quantification of segmentable brain structures [17–20], which combines two sequential pipelines. (1) An automated method for white matter lesion segmentation that uses 3D T1-weighted and FLAIR MR images in a probabilistic model. The accuracy and reproducibility of this software has been shown to be comparable to other well-established MS lesion segmentation algorithms (e.g., Lesion-TOADS, Lesion Segmentation Tool [17, 18]); (2) An automated multi-atlas cortical and subcortical segmentation method, which showed similar accuracy and reproducibility to commonly used automatic segmentation tools [21, 22]. Icobrain ms allowed extraction of normalized volumes for 34 anatomical regions, including cerebral gray matter regions (*n* = 20), deep gray matter regions (*n* = 7), brainstem (*n* = 3), cerebellum (*n* = 2), lateral ventricles (*n* = 1), and cerebral white matter (*n* = 1). In addition, icobrain ms allowed calculation of the corpus callosum area (*n* = 1) and upper cervical cord area (*n* = 1). As the cervical spinal cord was within the field of view on brain scans, the mean upper cervical cord area along C2–C3 levels was computed from the brain images [23, 24] using the icobrain ms fully automated pipeline [25, 26] based on the Spinal Cord Toolbox [27] (see Table 1 for more details). After visual inspection of the segmentation output, no participants were excluded from the analyses due to low quality MRI data or unsuccessful segmentation outputs.

### Standard clinical measures

During three clinical visits (baseline, week 12, and week 24) PwMS underwent clinical evaluation. These included the oral Symbol Digital Modalities Test (SDMT), Nine-Hole Peg Test (9HPT), Berg Balance Scale, Timed 25-Foot Walk (T25FW), and EDSS.

### Statistical analysis

Due to the exploratory nature of this analysis, an unbiased approach with no pre-specified hypotheses was adopted to investigate associations between the brain/spinal cord regions and the digital measures obtained with the Floodlight PoC app (Table 1) and the standard clinical measures. Two separate cross-sectional analyses were conducted on the digital measures and, for comparison, on the standard clinical measures. First, these measures were correlated against regional, structural MRI outcomes using Spearman’s rank correlation. Since the digital measures may be confounded by age, sex, and body mass index, the correlation analysis was adjusted for these confounders with a robust linear model via iteratively reweighted least squares [28]. Statistical significance was set at *q* < 0.05 after applying the false discovery rate (FDR) correction to correct for multiple comparisons for each digital and standard clinical measure separately [29].

In the second analysis, the variance in the digital measures that can be explained by volumetric MRI data was estimated. Here, a Bayesian ridge regression model [30] with leave-one-out cross validation was used to estimate the *R*^2^ score, i.e., a measure related to the proportion of the variance in the dependent variable (digital measure or standard clinical measure) that is predictable from the independent variables (structural MRI outcomes). The following two models were considered: (1) the univariate Whole Brain model, which included the normalized total brain volume to estimate the variance in the digital measures and standard clinical measures; (2) the multivariate Parcellation model, which instead included the normalized volume and cross-sectional area measurements of 36 MRI regions.

Given the relatively short duration of the study (24 weeks) and the stability of the clinical and MRI measures over the study period (Fig. SI1), a data aggregation approach was followed to reduce variability and to deal more effectively with missing data. For both analyses, the data were aggregated as follows: for the digital measures, the median across all valid active tests was calculated; for the standard clinical measures and MRI outcomes, the mean of the three clinical visits (baseline, week 12, and week 24) and two MRI scans (baseline and week 24), respectively, were calculated.

All statistical analyses were performed in Python 3.7.9 (www.python.org) using statsmodels 0.12.0, an open-source module for data analysis and scientific computing (www.statsmodels.org).

## Results

The study enrolled 76 PwMS between November 28, 2016 and November 13, 2017 (study completion date was May 4, 2018), of which 62 (82%) PwMS were included in the analyses presented here, comprising of 11 PwMS from the University of California San Francisco and 51 PwMS from the Hospital Universitari Vall d'Hebron. Of the 14 excluded PwMS, 12 were excluded for poor adherence to the active tests and two for missing MRI scans. Full baseline demographics and disease characteristics have been previously published [14] and those included in the current analyses are provided in Table 2. Of those PwMS included, 68% were female, mean age was 39.7 years (standard deviation [SD] 7.5), mean EDSS at baseline was 2.5 (SD 1.4; range 0.0–5.5), and 89% were diagnosed with relapsing–remitting MS. In order to exclude any potential effect of the MS phenotype on volumetric MRI data, we compared all 36 regions of interest between the groups of people with relapsing–remitting MS and progressive MS. As reported in Fig. SI2, significant differences were observed in only one of the 36 regions (combined occipital regions of cuneus, lingual gyrus, and pericalcarine cortex). This suggests that regional volumes were not dissimilar across relapsing and progressive MS phenotypes in this cohort. Additionally, no significant changes between the two study sites were found for age (*p* = 0.846), sex (*p* = 0.145), body mass index (*p* = 0.905), and disease phenotype (*p* = 0.436) (Fig. SI3).

**Table 2:** Baseline demographics and disease characteristics

Variable	PwMS cohort included in the analyses (*n* = 62)
Site	11 (USA), 51 (Spain)
Female, *n*/*N* (%)	42/62 (67.7)
Age, years, mean (SD)	
Mean (SD)	39.7 (7.5)
Min.–Max.	20–57
BMI, kg/m^2^	
Mean (SD)	24.4 (4.4)
Min.–Max.	17.1–37.6
Diagnosis, *n* (%)	
RRMS	55 (89)
PPMS	3 (5)
SPMS	4 (6)
Disease duration at baseline, years	
Mean (SD)	9.5 (6.6)
Min.–Max.	0.7–24.9
Normalized total brain volume, mL	
Mean (SD)	1471.9 (65.0)
Min.–Max.	1277.1–1628.1
EDSS	
Mean (SD)	2.5 (1.4)
Min.–Max.	0.0–5.5
Oral SDMT, number of correct responses	
Mean (SD)	53.4 (11.7)
Min.–Max.	26–77
9HPT, seconds	
Mean (SD)	22.4 (4.3)
Min.–Max.	16.4–40.3
T25FW, seconds	
Mean (SD)	6 (2)
Min.–Max.	3.6–12.5
Berg Balance Scale, total score	
Mean (SD)	52.4 (5.8)
Min.–Max.	31–56

*9HPT* Nine-Hole Peg Test, *BMI* body mass index, *EDSS* Expanded Disability Status Scale, *PPMS* primary progressive multiple sclerosis, *PwMS* people with multiple sclerosis, *RRMS* relapsing–remitting multiple sclerosis, *SD* standard deviation, *SDMT* Symbol Digit Modalities Test, *SPMS* secondary progressive multiple sclerosis, *T25FW* Timed 25-Foot Walk

**Fig. 2:**
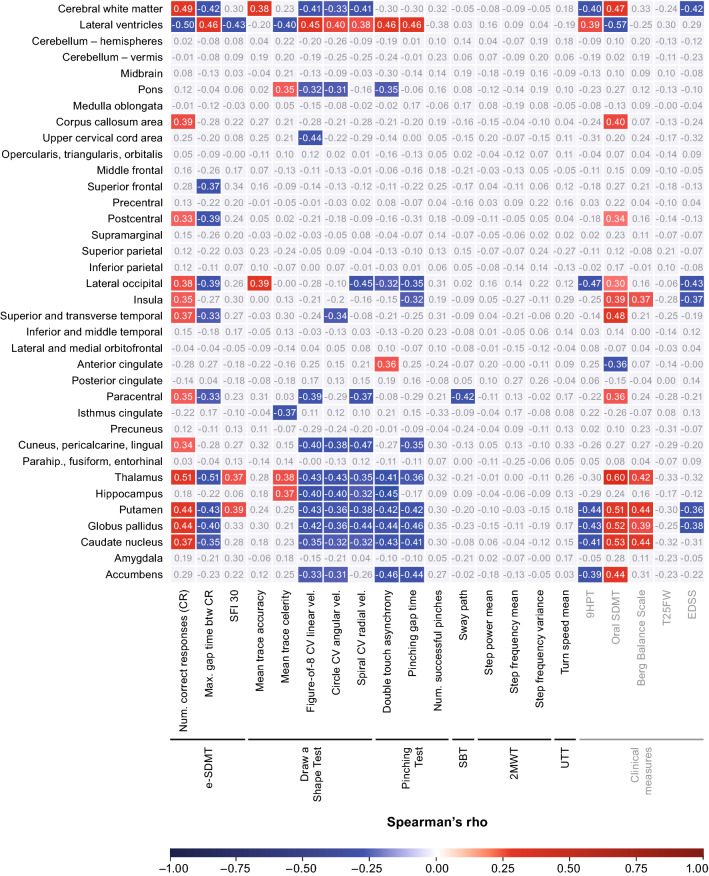
Spearman’s rank correlation analysis of digital measures and standard clinical measures with global and regional MRI outcomes. Statistically significant (*q* < 0.05) positive and negative correlations are highlighted in red and blue, respectively. FDR correction was applied for each digital and standard clinical measure separately to correct for multiple comparisons. Higher values equate to better performance on the oral SDMT and Berg Balance Scale, as well as for digital measures assessing number of correct responses and SFI 30 on the e-SDMT; trace accuracy and trace celerity on the Draw a Shape Test; number of pinches on the Pinching Test; mean step power and mean step frequency on the 2MWT; and mean turn speed on the UTT. In contrast, higher values equate to worse performance on the EDSS, 9HPT, and T25FW, as well as for digital measures assessing max. gap time between correct responses on the e-SDMT; CV linear, angular, and radial velocity on the Draw a Shape Test; double touch asynchrony and pinching gap time on the Pinching Test; sway path on the SBT; and step frequency variance on the 2MWT. *2MWT* Two-Minute Walk Test, *9HPT* Nine-Hole Peg Test, *btw* between, *CR* correct responses, *CV* coefficient of variation, *EDSS* Expanded Disability Status Scale, *e-SDMT* smartphone-based electronic Symbol Digit Modalities Test, *FDR* false discovery rate, *num.* number of, *parahip.* parahippocampus, *SBT* Static Balance Test, *SDMT* Symbol Digit Modalities Test, *SFI* speed fatigability index, *T25FW* Timed 25-Foot Walk, *UTT* U-Turn Test, *vel.* drawing velocity

**Fig. 3:**
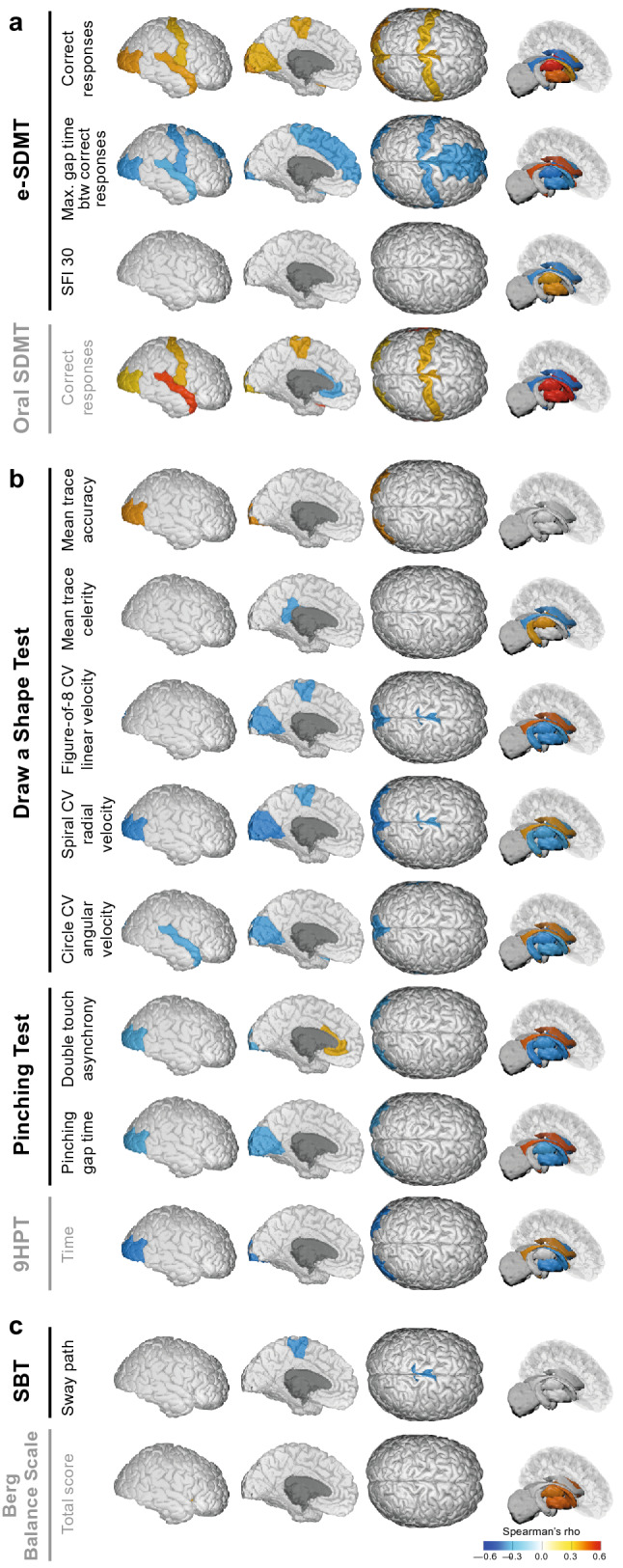
Spearman’s rank correlations adjustment for age, sex, and BMI. Statistically significant (*q* < 0.05) correlations between regional brain volume measured by MRI and measures of **a** the cognitive domain, **b** upper extremity function domain, and **c** balance domain are shown for four planes (from left to right: cortical outer, inner, top, and subcortical). FDR correction was applied for each digital and standard clinical measure separately to correct for multiple comparisons. Higher values equate to better performance for digital measures assessing number of correct responses and SFI 30 on the e-SDMT; trace accuracy and trace celerity on the Draw a Shape Test; and double touch asynchrony on the Pinching Test. In contrast, higher values equate to worse performance for digital measures assessing max. gap time between correct responses on the e-SDMT; CV linear, angular, and radial velocity on the Draw a Shape Test; pinching gap time on the Pinching Test; and sway path on the SBT. *9HPT* Nine-Hole Peg Test, *BMI* body mass index, *btw* between, *CV* coefficient of variation, *e-SDMT* smartphone-based electronic Symbol Digit Modalities Test, *FDR* false discovery rate, *SBT* Static Balance Test, *SDMT* Symbol Digit Modalities Test, *SFI* speed fatigability index

The regional neural correlates of the digital measures and the standard clinical measures are summarized in Fig. 2. Uncorrected *p* values and the respective FDR-corrected *p* values (*q* values) are reported in the supplementary appendix (Fig. SI4 and SI5). Brain maps highlighting the statistically significant correlations after FDR correction are provided in Fig. 3. Higher EDSS scores correlated with smaller volumes of the cerebral white matter (*r* =  − 0.42, *q* < 0.05), lateral occipital lobe (*r* =  − 0.43, *q* < 0.05), insula (*r* =  − 0.37, *q* < 0.05), putamen (*r* =  − 0.36, *q* < 0.05), and globus pallidus (*r* =  − 0.38, *q* < 0.05).

The e-SDMT and oral SDMT showed a similar correlation pattern with regional MRI outcomes (Figs. 2, 3a). On both, a lower number of correct responses was significantly associated with smaller volumes of the cerebral white matter (*r* = 0.49 for e-SDMT/*r* = 0.47 for oral SDMT), corpus callosum (*r* = 0.39/0.40, *q* < 0.05), postcentral gyrus (*r* = 0.33/0.34, *q* < 0.05), lateral occipital lobe (*r* = 0.38/0.30, *q* < 0.05), insula (*r* = 0.35/0.39, *q* < 0.05), superior and transverse temporal gyrus (*r* = 0.37/0.48, *q* < 0.05), paracentral gyrus (*r* = 0.35/0.36, *q* < 0.05), and deep gray matter structures such as the thalamus, putamen, globus pallidus, and caudate nucleus (*r* = 0.37–0.51/0.51–0.60, *q* < 0.05). A lower number of correct responses on either test was also associated with a larger volume of the lateral ventricles (*r* =  − 0.50/ − 0.57, *q* < 0.05). Two correlations were only observed on the e-SDMT, but not on the oral SDMT. This included the correlation between the e-SDMT number of correct responses and the volume of the cuneus, pericalcarine cortex, lingual gyrus (*r* = 0.34, *q* < 0.05), and between the e-SDMT maximum gap duration between correct responses and the volume of the superior frontal lobe (*r* =  − 0.37, *q* < 0.05).

Likewise, for each regional MRI outcome that showed significant correlation with the 9HPT there was at least one Draw a Shape Test measure that was significantly correlated with the same MRI outcome (Figs. 2, 3b, SI6a). Worse performance on either test was associated with smaller volumes of the cerebral white matter (│*r*│ = 0.33–0.41, *q* < 0.05), lateral occipital lobe (│*r*│ = 0.39–0.47, *q* < 0.05), and deep gray matter nuclei such as the putamen, globus pallidus, caudate nucleus, and accumbens (│*r*│ = 0.31–0.44, *q* < 0.05), as well as a larger volume of the lateral ventricles (│*r*│ = 0.38–0.45, *q* < 0.05). However, some correlates were only observed on the Draw a Shape Test, but not the 9HPT. For example, mean trace celerity correlated with volumes of the thalamus (*r* = 0.38, *q* < 0.05), hippocampus (*r* = 0.37, *q* < 0.05), and isthmus cingulate (*r* =  − 0.37, *q* < 0.05). Velocity-based measures of round shapes correlated with volumes of the thalamus (│*r*│ = 0.35–0.43, *q* < 0.05), hippocampus (│*r*│ = 0.32–0.40, *q* < 0.05), and cuneus, pericalcarine cortex, and lingual gyrus (│*r*│ = 0.38–0.47, *q* < 0.05). A significant association was also observed between variability of linear drawing velocity on the figure-of-8 and the cervical spinal cord area (*r* =  − 0.44, *q* < 0.05).

Similarly, for each correlation observed with the 9HPT, at least one Pinching Test measure was significantly associated with the same MRI outcome, except for the cerebral white matter (Figs. 2, 3b). Some correlations, however, were observed only with the Pinching Test. This includes correlations between longer double touch asynchrony and smaller volumes of the pons (*r* =  − 0.35, *q* < 0.05), thalamus (*r* =  − 0.41, *q* < 0.05), hippocampus (*r* =  − 0.45, *q* < 0.05), and interestingly with a larger volume of the anterior cingulate cortex (*r* = 0.36, *q* < 0.05). Additionally, longer gap duration between pinches was also associated with a smaller volume of the thalamus (*r* =  − 0.36, *q* < 0.05), insula (*r* =  − 0.32, *q* < 0.05), and the cuneus, pericalcarine cortex, and lingual gyrus (*r* =  − 0.35, *q* < 0.05).

On the SBT, larger sway path correlated with a smaller volume of the paracentral lobule (*r* =  − 0.42, *q* < 0.05; Figs. 2, 3c). In contrast, a lower total score on the Berg Balance Scale (worse ability to balance) correlated mostly with smaller volumes of deep gray matter structures (*r* = 0.39 − 0.44 for correlations with *q* < 0.05; Figs. 2, 3c).

No correlations with any of the regional MRI outcomes were found for any of the gait tests, neither for the digital measures derived from the 2MWT/UTT nor for the T25FW (Fig. 2; all *q* ≥ 0.05).

**Fig. 4:**
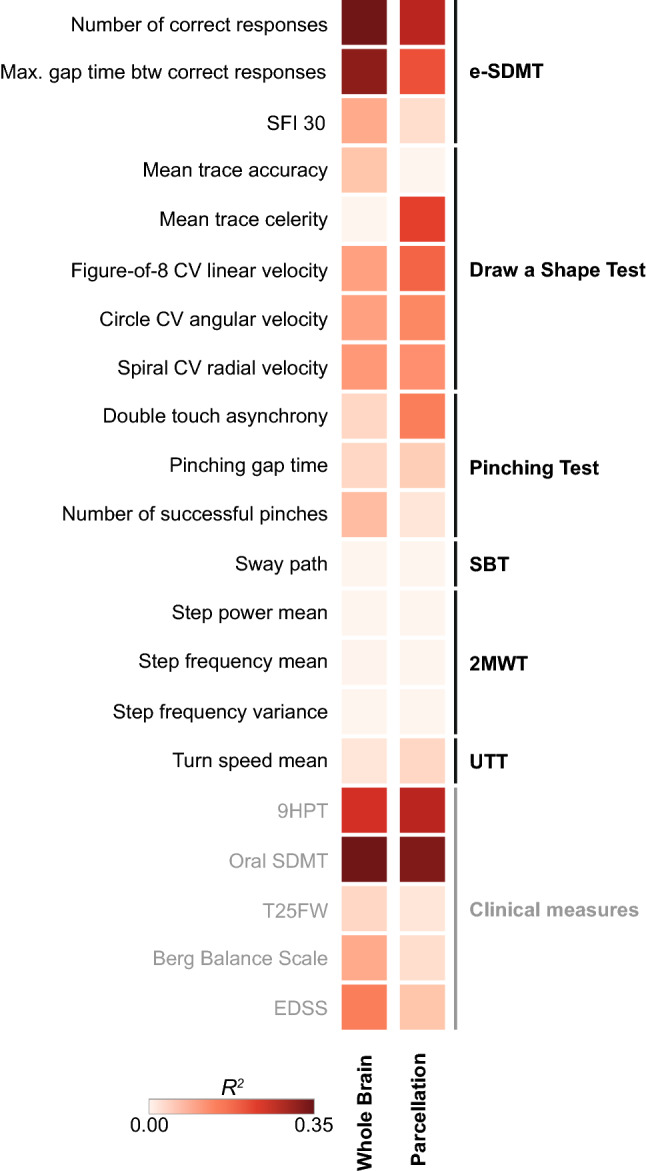
Bayesian ridge regression model with leave-one-out cross validation for estimating the variance (*R*^*2*^) in the digital measures and standard clinical measures that can be explained by volumetric MRI. Two models were applied. The first model (“Whole Brain”) included normalized brain volume and the three demographic variables: age, sex, and body mass index (top row). The second model (“Parcellation”) included all 36 regional MRI regions and the same three demographic variables (bottom row). Compared with the standard clinical measures, the digital measures tend to have a larger *R*^2^ score in the Parcellation model vs. in the Whole Brain model, which may reflect higher functional specificity. This is particularly evident on the Draw a Shape Test (mean trace celerity and spiral CV radial velocity) and on the Pinching Test (double touch asynchrony). *2MWT* Two-Minute Walk Test, *9HPT* Nine-Hole Peg Test, *btw* between, *CV* coefficient of variation, *EDSS* Expanded Disability Status Scale, *e-SDMT* smartphone-based electronic Symbol Digit Modalities Test, *SBT* Static Balance Test, *SDMT* Symbol Digit Modalities Test, *SFI* speed fatigability index, *T25FW* Timed 25-Foot Walk, *UTT* U-Turn Test

Next, the variance observed in the digital measures that can be explained by structural MRI outcomes was estimated using a Bayesian ridge regression model. Up to a third of the variance observed in the digital measures (*R*^2^ = 34%) could be explained when using either normalized total brain volume as individual predictor (“Whole Brain” model) or volumetric data from the 36 individual MRI regions as multiple predictors (“Parcellation” model). In the upper extremity function domain, however, comparable or higher *R*^*2*^ values were obtained with the Parcellation model compared with the Whole Brain model. This suggests that multiple predictors, thus multiple brain regions, can explain as much or more of the observed variance in the digital measures (Figs. 4, SI6b). The increase in *R*^2^ was most noticeable for mean trace celerity (*R*^2^ = 0.23 vs. 0), spiral coefficient of variation (CV) linear drawing velocity (*R*^2^ = 0.22 vs. 0.02), figure-of-8 CV linear drawing velocity (*R*^2^ = 0.19 vs. 0.12), and double-touch asynchrony (*R*^2^ = 0.16 vs. 0.06, respectively, for the Parcellation vs. Whole Brain model). This was also reflected by a less uniform distribution of the individual Bayesian ridge regression coefficients across the 36 MRI regions (Fig. SI7). By comparison, the *R*^*2*^ score was more comparable across the two models for the standard clinical measure 9HPT (*R*^2^ = 0.28 vs. 0.25) (Fig. 4).

## Discussion

Digital measures captured by the Floodlight PoC app demonstrated robust correlations with volumetric measurements of specific brain regions. While many neural correlates were shared between clinical and digital measures, the latter showed associations with brain regions that clinical measures were unable to demonstrate. These results indicate that remotely administered digital tests may yield higher functional specificity and provide potential novel information on functional ability compared with standard clinical measures. Of note, these correlations were observed in an early, relapsing cohort of PwMS, suggesting that digital measures hold potential to capture silent pathology [31]. If confirmed in future work, this would address a key unmet need in MS disease management [32].

As expected, both the e-SDMT and oral SDMT correlated with regions implicated in cognitive decline (ventricular expansion and smaller volume of the insula and cerebral white matter), information processing speed (deep gray matter), and working memory (superior temporal gyrus) [33–39]. Correlations were also observed with the volume of the primary somatosensory cortex (postcentral gyrus) and visual processing areas (lateral occipital lobe) [34, 40]. Similarly, the Draw a Shape Test, Pinching Test, and 9HPT were associated with areas of visual processing (lateral occipital lobe, deep gray matter) and cognitive decline (ventricular expansion, cerebral white matter) [34–36]. For the SBT, postural control (sway path) correlated inversely with the paracentral lobule volume. This is consistent with findings from a functional imaging study, which suggested a role of the paracentral lobule in proprioceptive processing [41]. In contrast, the Berg Balance Scale correlated more strongly with the deep gray matter volume. The different tasks involved in the two tests, the distinctive sensorimotor aspects they capture, and the scoring—the SBT measures the total sway path (a measure of static balance), while the Berg Balance Scale provides an overall score describing both static and dynamic balance—may explain these differences. The mild level of MS-related impairment together with the ceiling effect observed on the Berg Balance Scale may have contributed to the lack of correlations with the cerebellar volume. The former may also explain the lack of correlations found for the sensor-based walking tests (UTT, 2MWT) or the T25FW [42]. With greater levels of impairment, correlations between the T25FW and thalamic volumes or upper cervical cord area could be expected [43, 44].

Several correlations were only observed for the digital measures, but not for the standard clinical measures. This was most evident in the upper extremity function domain. Temporal and spatiotemporal measures derived from the Draw a Shape Test such as the mean trace celerity and variability of drawing velocity while drawing round shapes specifically correlated with thalamic volume, which has been previously shown to be involved in information processing [45]. These measures also specifically correlated with the volume of the pons. Brainstem atrophy is reported in early stages of MS and a higher pontine lesion load has been associated with upper extremity tremor [7, 46]. In addition, the variability of linear drawing velocity on the figure-of-8 specifically correlated with the upper cervical cord area. This region has been previously linked to upper extremity dysfunction, particularly in more advanced or progressive disease where atrophy is more pronounced [44, 47]. The fact that we observed this correlation in a mildly impaired cohort highlights the potential higher sensitivity of Floodlight PoC digital measures. Correlations specific to the digital measures were also observed with the Pinching Test. Double touch asynchrony, that corresponds to an asynchronous contact of the two fingers with the touchscreen while pinching a tomato, was associated with the volume of anterior cingulate cortex. No association was observed with the 9HPT time. This region is known to selectively modulate motor areas during visually coordinated tasks [48, 49]. This result is not surprising considering that double touch asynchrony was specifically developed to assess the ability to perform finger coordination tasks.

The notion that digital measures may offer higher functional specificity is also supported by the explained variance analysis. A low *R*^*2*^ value in the Whole Brain model but a high *R*^*2*^ value in the Parcellation model indicates that certain regions contribute more than other regions to the variance observed in the digital or standard clinical measures. This can be seen in the upper extremity function domain, where selected measures derived from the Draw a Shape and Pinching Tests showed a larger increase in *R*^*2*^ when switching from the Whole Brain to the Parcellation model compared with the 9HPT. This suggests that these digital measures have higher functional specificity than the 9HPT. A high *R*^2^ value in both models, on the other hand, indicates that the different MRI regions contribute equally to the observed variance. The cognitive domain with both the e-SDMT and oral SDMT is a good example of this, with both showing comparable functional specificity.

There are some limitations to this study. First, MRI data were only collected for PwMS. Consequently, we were unable to disentangle physiologic from pathologic effects. Second, the relatively short study duration of 24 weeks did not allow us to perform longitudinal analyses or assess the relationship between the digital measures and disease progression. Third, PwMS enrolled in this study mostly had mild disease with limited functional impairment, which may have weakened the correlations. Structural, or even functional, cortical reorganization, that helps to maintain functional ability in early stages of the disease despite structural damage to the brain [50–53], may have further weakened the observed correlations. Finally, spurious correlations cannot be excluded. However, the observed effects remained largely statistically significant even when applying a more conservative correction for multiple testing (FDR correction applied for all the possible combinations of the 36 anatomical regions and 21 digital/clinical measures [36 × 21 configuration]), suggesting that potential spurious correlations are negligible (Fig. SI8).

In the future, using data from larger, ongoing, and forthcoming studies (CONSONANCE, NCT03523858; Floodlight™ MS—TONiC, ISRCTN11088592), we will explore both cortical and subcortical structural networks [54, 55] and assess the relationship between longitudinal changes in digital measures and MRI outcomes to better characterize the utility of sensor-based tests as prognostic biomarkers. Such biomarkers could be used for early identification of patients with silent progression at risk of future disability accrual, and optimization of individual treatment strategies.

## Conclusions

In this exploratory post-hoc analysis, digital measures obtained with the Floodlight PoC app correlated with normalized volumes of distinct anatomical regions. While many of the correlations were also observed with standard clinical measures, some were only observed with the digital measures. In addition, the explained variance analysis may suggest a higher functional specificity for digital measures, in particular in the upper extremity function domain. These results indicate that digital measures, by leveraging sensor technology, can probe multiple different neurologic domains rather than just providing an overall assessment of functional ability. Thus, digital measures have the potential to complement standard clinical measures by providing a more detailed picture of MS and a more accessible assessment of functional ability. Larger, ongoing, and forthcoming studies will need to confirm these preliminary findings.

## Supplementary Information

Below is the link to the electronic supplementary material.

Supplementary file1 (PDF 9479 KB)

## Data Availability

For up-to-date details on Roche's Global Policy on Sharing of Clinical Study Information and how to request access to related clinical study documents, see here: https://go.roche.com/data_sharing. Request for the data underlying this publication requires a detailed, hypothesis-driven, statistical analysis plan that is collaboratively developed by the requestor and company subject matter experts. Such requests should be directed to dbm.datarequest@roche.com for consideration. Anonymized records for individual patients across more than one data source external to Roche cannot, and should not, be linked due to a potential increase in risk of patient re-identification.
