# Supplementary material for: Neural correlates of digital measures shown by structural MRI: a post-hoc analysis of a smartphone-based remote assessment feasibility study in multiple sclerosis
Source: J Neurol. 2022 Dec 5;270(3):1624–36. doi: 10.1007/s00415-022-11494-0 (PMC9970954; doi:10.1007/s00415-022-11494-0)
Supplement: Supplementary file 1 — Supplementary file1 (PDF 9479 KB) [file 415_2022_11494_MOESM1_ESM.pdf]

Supplementary appendix

Journal of Neurology

Neural correlates of digital measures shown by structural MRI: a post-hoc analysis of a smartphone-based remote assessment feasibility study in multiple sclerosis

Marco Ganzetti[1] · Jennifer S. Graves[2] · Sven P. Holm[1] · Frank Dondelinger[1*] · Luciana Midaglia[3,4] · Laura Gaetano[1] · Licinio Craveiro[1] · Florian Lipsmeier[1] · Corrado Bernasconi[1] · Xavier Montalban[3,4] · Stephen L. Hauser[5] · Michael Lindemann[1]

[1] F. Hoffmann-La Roche Ltd, Basel, Switzerland

[2] Department of Neurosciences, University of California San Diego, San Diego, CA, USA

[3] Department of Neurology-Neuroimmunology, Centre d'Esclerosi Múltiple de Catalunya (Cemcat), Hospital Universitari Vall d'Hebron, Barcelona, Spain

[4] Department of Medicine, Autonomous University of Barcelona, Barcelona, Spain

[5] Department of Neurology, University of California San Francisco, San Francisco, CA, USA

* At the time of writing author was an employee of F. Hoffmann-La Roche Ltd; current affiliation is Novartis Institutes for Biomedical Research, Basel, Switzerland

Correspondence

Dr. Florian Lipsmeier – F. Hoffmann-La Roche Ltd, Basel, Switzerland

florian.lipsmeier@roche.com

Table SI1 Computational definitions of the digital measures

Test	Feature	Computational definition	Variables
Draw a Shape Test	*Mean trace accuracy*	$\frac{1}{N}\sum_{i=1}^{N} DT_i \cap RT_i$	DT_i: touch points drawn trace of shape i<br>RT_i: reference trace of shape i<br>N: number of drawn shapes during an active test (max. 6)
	Mean trace celerity, 1/s	$\frac{1}{N}\sum_{i=1}^{N} \frac{TA_i}{T_i}$	TA_i: trace accuracy for shape i<br>T_i: drawing duration for shape i (s)<br>N: number of drawn shapes during an active test (max. 6)
	CV linear velocity[a]	$\frac{SD(\dot{y}(t_n))}{mean(\dot{y}(t_n))}$	$\dot{y}(t_n) = \frac{dy(t)}{dt}\Big\vert_{t=t_n}$*:* derivative of the displacement (mm/s)<br>$y(t)$ *:* the displacement time series as a function of time
	CV angular velocity[b]	$\frac{SD\left(\dot{\Theta}(t_n)\right)}{mean\left(\dot{\Theta}(t_n)\right)}$	$\dot{\Theta}(t_n) = \frac{d\Theta(t)}{dt}\Big\vert_{t=t_n}$*:* derivative of the angular displacement (rad/s)<br>$\Theta(t)$ *:* the angular displacement time series as a function of time
	CV radial velocity[b]	$\frac{SD(\dot{r}(t_n))}{mean(\dot{r}(t_n))}$	$\dot{r}(t_n) = \frac{dr(t)}{dt}\Big\vert_{t=t_n}$*:* derivative of the radial displacement (mm/s)<br>$r(t)$ *:* the radial displacement time series as a function of time
Pinching Test	*Number of successful pinches, n*	$\sum_{i=1}^{N}(SP_i)$	$SP_i = \begin{cases} 1\ if\ pinch\ attempt_i\ was\ successful \\ 0\ if\ pinch\ attempt_i\ was\ not\ successful \end{cases}$<br>$N$: number of pinch attempts during an active test
	Gap time between pinch attempts, s	$\frac{1}{N}\sum_{i=2}^{N}(B_i - E_{i-1})$	B_i: beginning of attempt (first finger touches the screen) i (s)<br>E_{i-1}: end of attempt (last finger leaves the screen) i–1 (s)<br>N: number of pinch attempts during an active test
	Double touch asynchrony, s	$\frac{1}{N}\sum_{i=1}^{N}(t_{f2i} - t_{f1i})$	t_{f1i}: time at which the first finger touches the screen for attempt i (s)<br>t_{f2i}: time at which the second finger touches the screen for attempt i (s)<br>N: number of pinch attempts during an active test
e-SDMT	*Number of correct responses, n*	$\sum_{i=1}^{N}(CR_i)$	$CR_i = \begin{cases} 1\ if\ response_i\ is\ correct \\ 0\ if\ response_i\ is\ incorrect \end{cases}$<br>$N$: number responses given during an active test

	Max. gap time between correct responses, s	$max(\Delta CR)$	$\Delta CR = [CRT_i - CRT_{i-1}, for\ i = 2{:}N]$: gap times between correct responses<br>CRT_i: time point for correct response i (s)<br>N: number of correct responses during an active test
	Speed fatigability index for the last 30 seconds	$\frac{CR_{60-90}}{max\ (CR_{0-30}, CR_{30-60})}$	CR_{0-30}: number of correct responses in the first 30 seconds of the test<br>CR_{30-60}: number of correct responses from 30 to 60 seconds of the test<br>CR_{60-90}: number of correct responses in the last 30 seconds of the test
SBT	*Sway path, m/s²*	$\sum_{i=2}^{N}\sqrt{\left(ACC_{AP(i)} - ACC_{AP(i-1)}\right)^2 + \left(ACC_{ML(i)} - ACC_{ML(i-1)}\right)^2}$	ACC_{AP}: anterior–posterior component of acceleration<br>ACC_{ML}: medio-lateral component of acceleration<br>N: number of samples in the active test
UTT	*Turn speed, rad/s*	$\frac{1}{N}\sum_{i=1}^{N}\frac{\theta_i}{T_i}$	θ_i: turn angle for turn i (rad)<br>T_i: turning duration for turn i (s)<br>N: number of turns during an active test
2MWT	*Step frequency, Hz*	$\frac{1}{N}\sum_{i=1}^{N}\frac{1}{ST_i}$	ST_i: step duration for step i (s)<br>N: number of steps during an active test
	Step frequency variance, Hz²	$\frac{\sum_{i=1}^{N}\left(\frac{1}{ST_i} - \left(\frac{1}{N}\sum_{i=1}^{N}\frac{1}{ST_i}\right)\right)^2}{N-1}$	ST_i: step duration for step i (s)<br>N: number of steps during an active test
	Step power, m²/s³	$\frac{1}{N}\sum_{i=1}^{N}\int_{t\_start_i}^{t\_end_i}\left(\left(\lvert ACC\rvert - mean(\lvert ACC\rvert)\right)^2\right)$	$\lvert ACC\rvert$: magnitude of acceleration<br>t_start_i: start time of step i<br>t_end_i: end time of step i<br>N: number of steps during an active test

2MWT Two-Minute Walk Test, *CV* coefficient of variation, *e-SDMT* smartphone-based electronic Symbol Digit Modalities Test, *SBT* Static Balance Test, *SD* standard deviation, *UTT* U-Turn Test

[a] Computed separately for each shape included in the Draw a Shape Test

[b] Computed for the circle and spiral

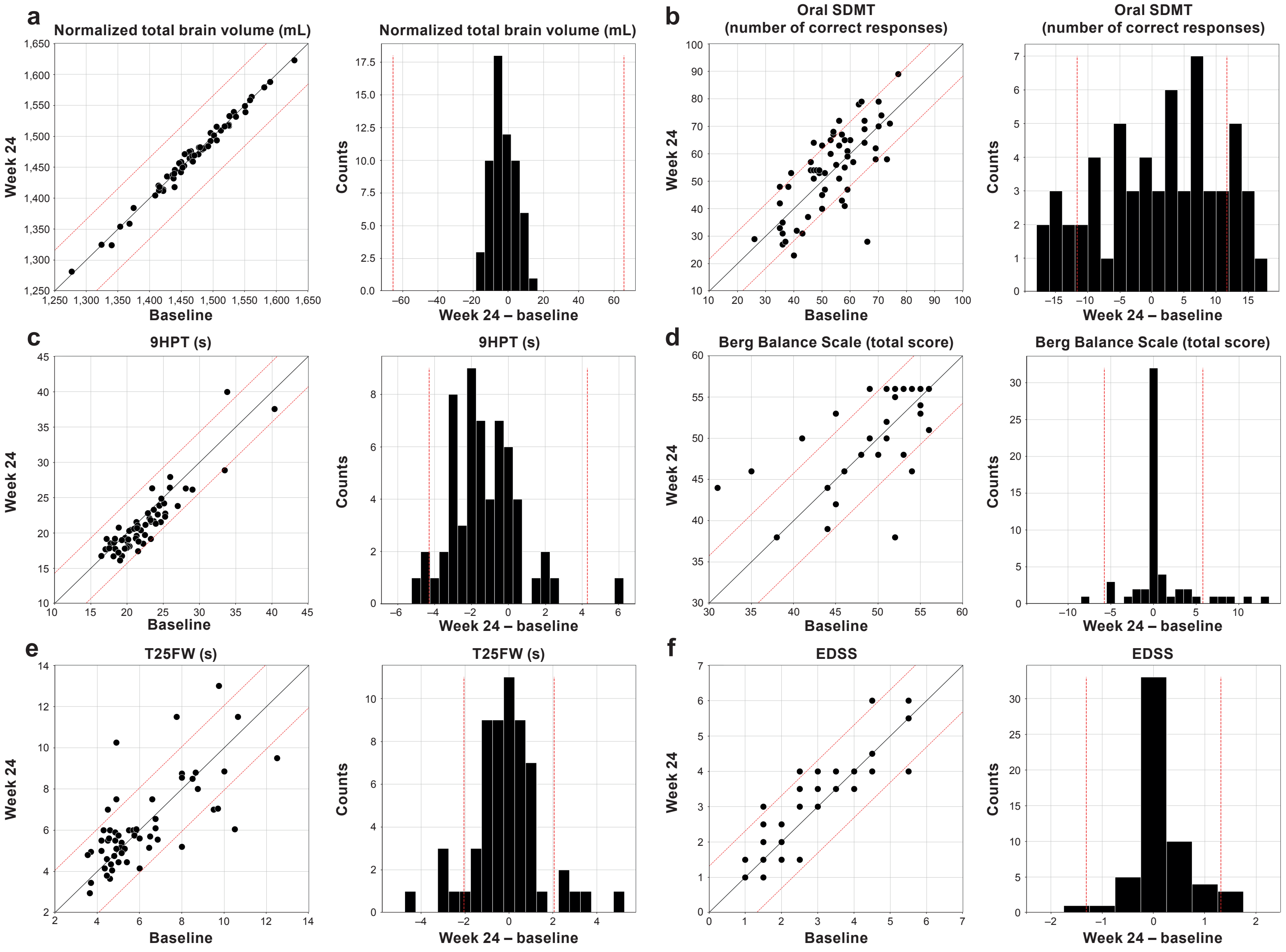

g

Variable	Number of participants	Mean (SD)	
		Baseline	Week 24
Normalized total brain volume, mL	61	1,471.75 (65.50)	1,471.18 (65.51)
Oral SDMT, number of correct responses	61	53.43 (11.68)	54.28 (15.22)
9HPT, s	59	22.41 (4.29)	21.36 (4.43)
Berg Balance Scale, total score	57	52.35 (5.75)	52.82 (5.19)
T25FW, s	60	6.06 (2.05)	6.16 (2.04)
EDSS	57	2.67 (1.31)	2.82 (1.36)

Fig. SI1 Clinical measures from baseline to week 24 (end of study)

Scatter plots and histograms showing the differences between baseline and week 24 (end of study) for **a** normalized total (whole) brain volume, **b** oral SDMT, **c** 9HPT, **d** Berg Balance Scale, **e** T25FW, and **f** EDSS. Changes observed on these clinical measures were mostly within one standard deviation of the baseline distribution (red dashed lines). **g** Mean and standard deviation for each of these clinical measures at both time points.
All subjects who had measures available for both the baseline and week 24 clinical visit were included.

9HPT Nine-Hole Peg Test, *EDSS* Expanded Disability Status Scale, *SDMT* Symbol Digit Modalities Test, *T25FW* Timed 25-Foot Walk

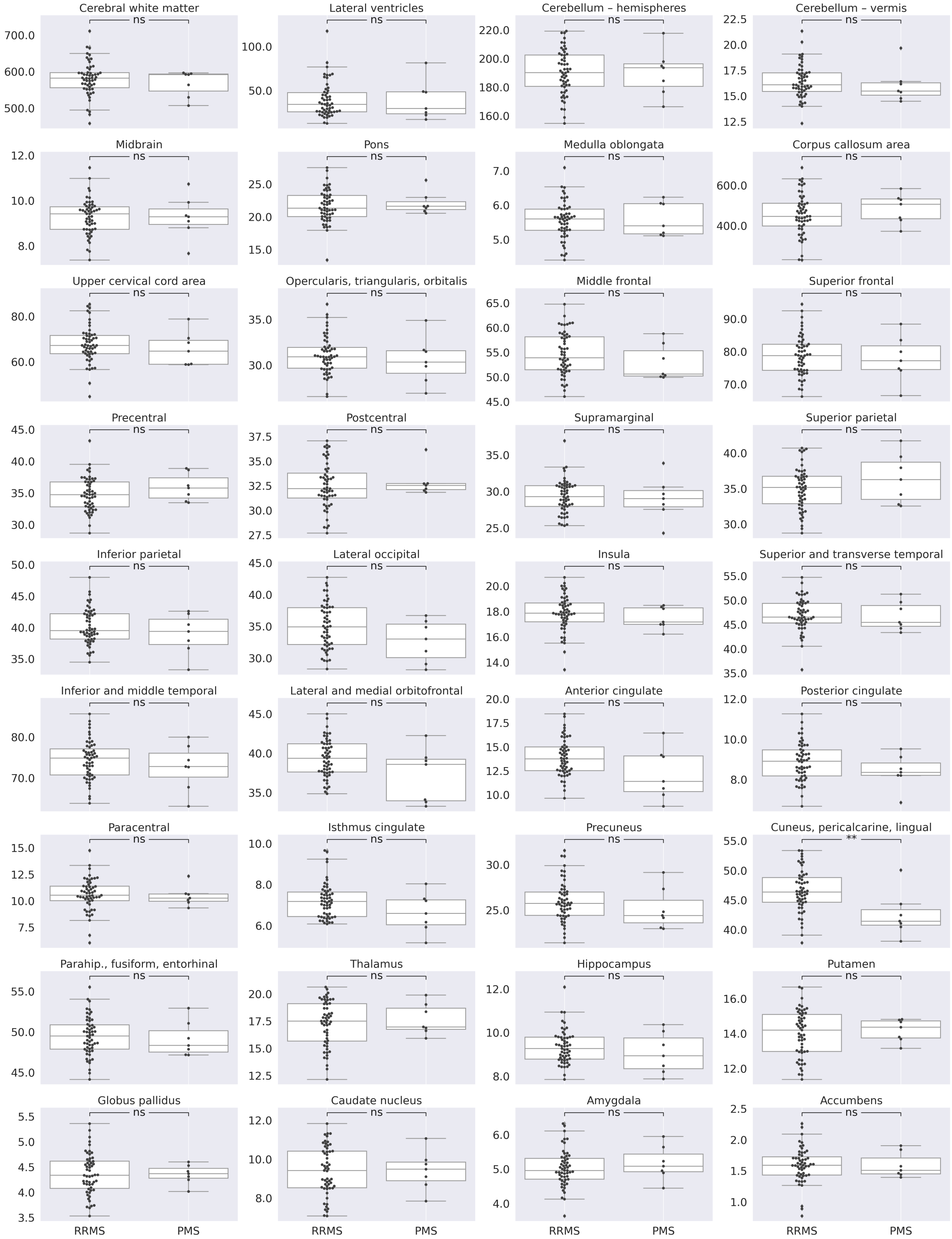

Fig. SI2 Normalized brain volume (mL) measures at baseline scan for the 36 anatomical regions included in the analysis, comparing people with RRMS and people with PMS

Please note that data for the corpus callosum and cervical spinal cord are areas expressed in mm^2 (not volumes).

***p*<0.01, *ns* not significant (Mann–Whitney U test), *parahip.* parahippocampus, *PMS* progressive multiple sclerosis, *RRMS* relapsing-remitting multiple sclerosis

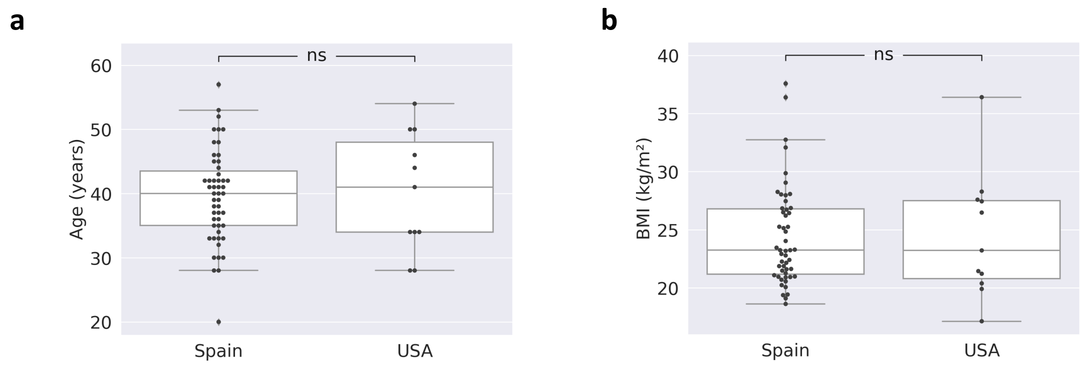

c

	Female	Male	Total
**Spain**	32	19	51
**USA**	10	1	11
**Total**	42	20	62

d

	RRMS	PMS	Total
**Spain**	44	7	51
**USA**	11	0	11
**Total**	55	7	62

Fig. SI3 Participant demographics by study site

a Age and **b** BMI values across the two study sites (University of California San Francisco, San Francisco, CA, USA and Multiple Sclerosis Centre of Catalonia, Vall d'Hebron University Hospital, Barcelona, Spain). There was no significant difference for age (Mann–Whitney U test, p=0.846) and BMI (Mann–Whitney U test, p=0.905) between study sites. **c** Sex distribution by study site (Chi-Square Test of Independence, p=0.145). **d** Disease phenotype distribution by study site (Chi-Square Test of Independence, p=0.436). People with primary and secondary PMS were pooled due to the small sample size of each individual cohort.

BMI body mass index, *ns* not significant, *PMS* progressive multiple sclerosis, *RRMS* relapsing-remitting multiple sclerosis

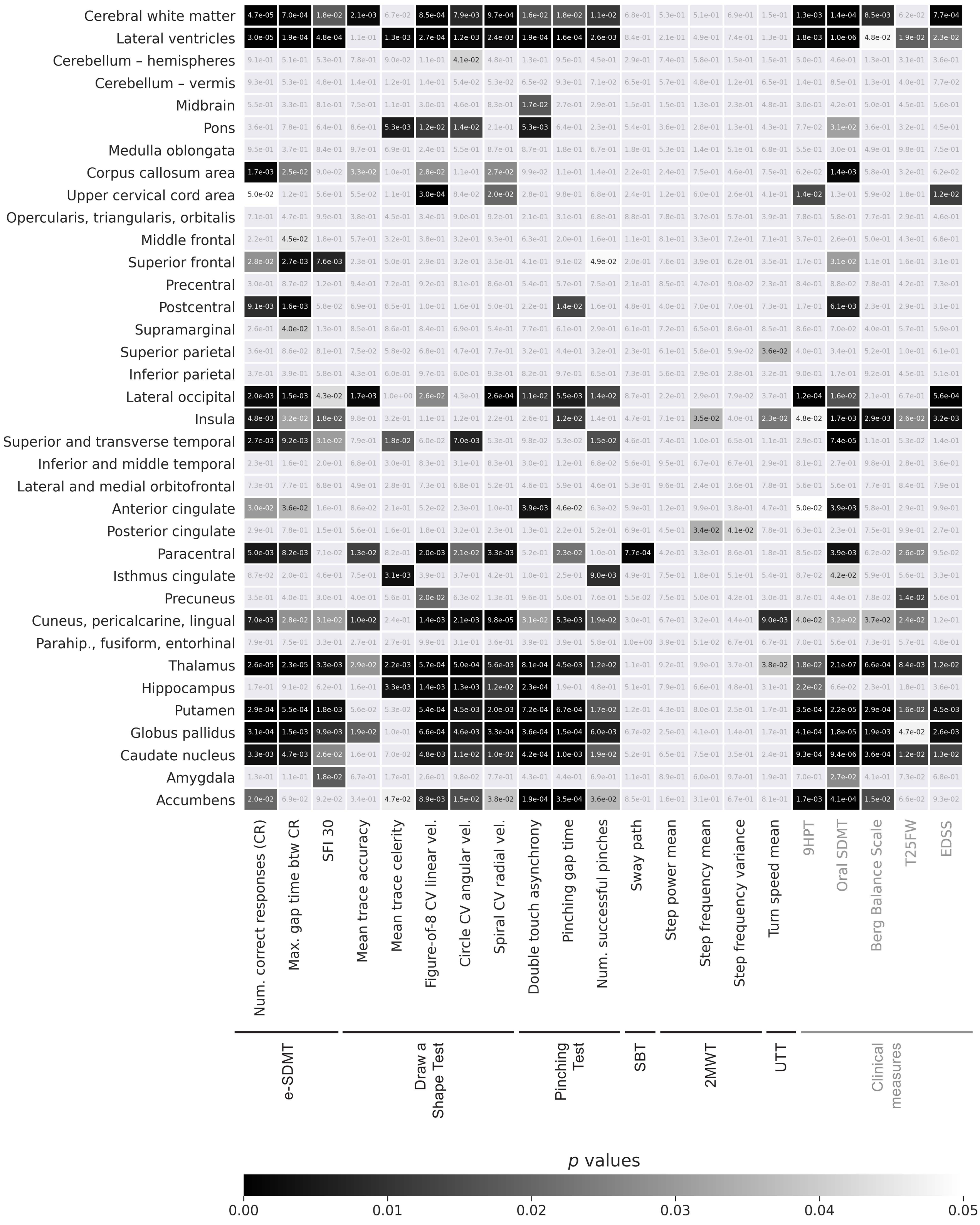

Fig. SI4 Uncorrected *p* values associated with the Spearman's rank correlation analysis of digital measures and standard clinical measures with global and regional MRI outcomes

Statistical significance ($p<0.05$) is highlighted in grayscale.

2MWT Two-Minute Walk Test, *9HPT* Nine-Hole Peg Test, *btw* between, *CR* correct responses, *CV* coefficient of variation, *EDSS* Expanded Disability Status Scale, *e-SDMT* smartphone-based electronic Symbol Digit Modalities Test, *max.* maximum, *num.* number of, *parahip.* parahippocampus, *SBT* Static Balance Test, *SDMT* Symbol Digit Modalities Test, *SFI* speed fatigability index, *T25FW* Timed 25-Foot Walk, *UTT* U-Turn Test, *vel.* drawing velocity

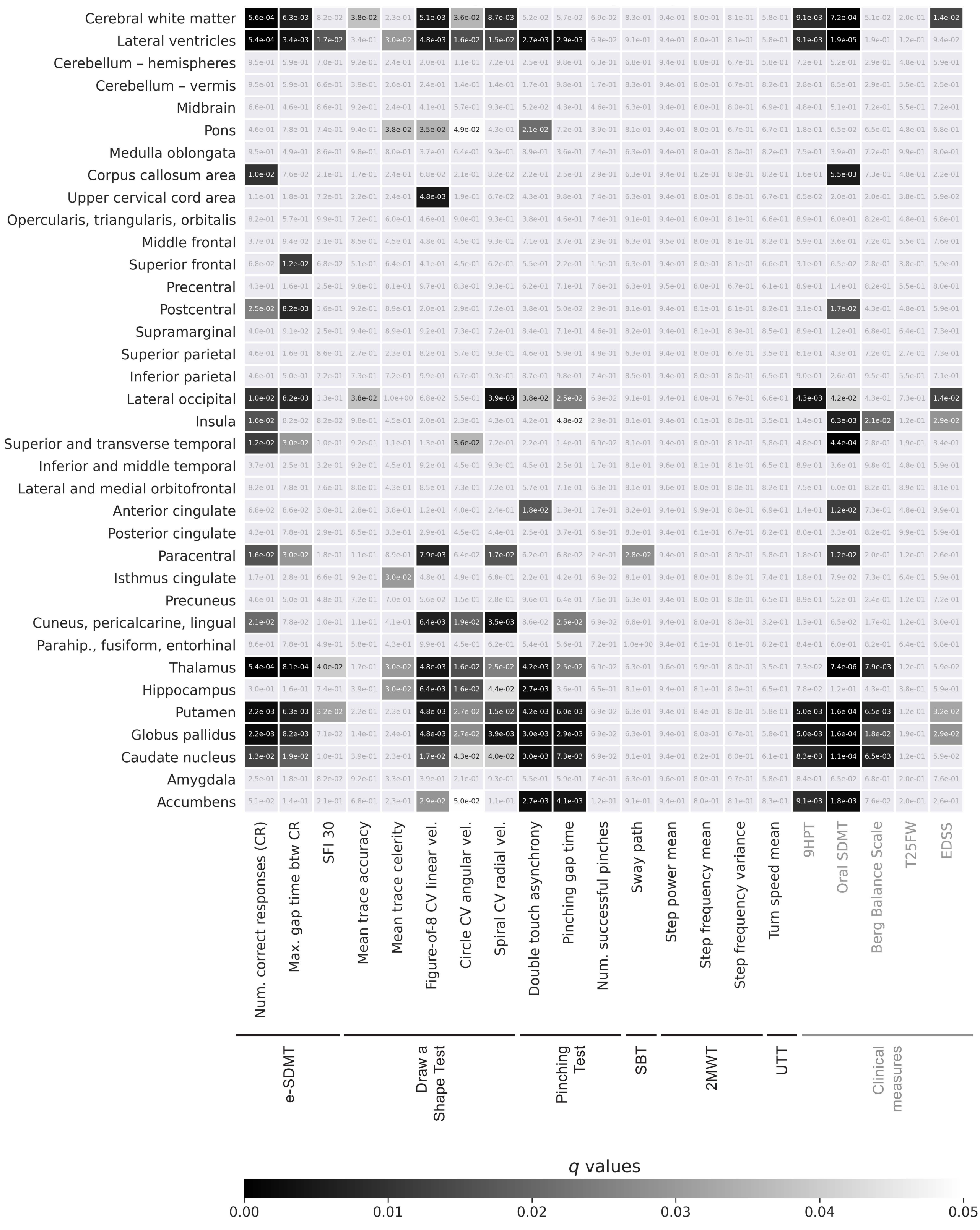

Fig. SI5 FDR-corrected *p* values (*q* values) associated with the Spearman's rank correlation analysis of digital measures and standard clinical measures with global and regional MRI outcomes

Statistical significance ($q<0.05$) is highlighted in grayscale. FDR correction is applied independently for each digital measure and clinical measure (36 [MRI outcomes] × 1 [digital measure] configuration).

2MWT Two-Minute Walk Test, *9HPT* Nine-Hole Peg Test, *btw* between, *CR* correct responses, *CV* coefficient of variation, *EDSS* Expanded Disability Status Scale, *e-SDMT* smartphone-based electronic Symbol Digit Modalities Test, *FDR* false discovery rate, *max.* maximum, *num.* number of, *parahip.* parahippocampus, *SBT* Static Balance Test, *SDMT* Symbol Digit Modalities Test, *SFI* speed fatigability index, *T25FW* Timed 25-Foot Walk, *UTT* U-Turn Test, *vel.* drawing velocity

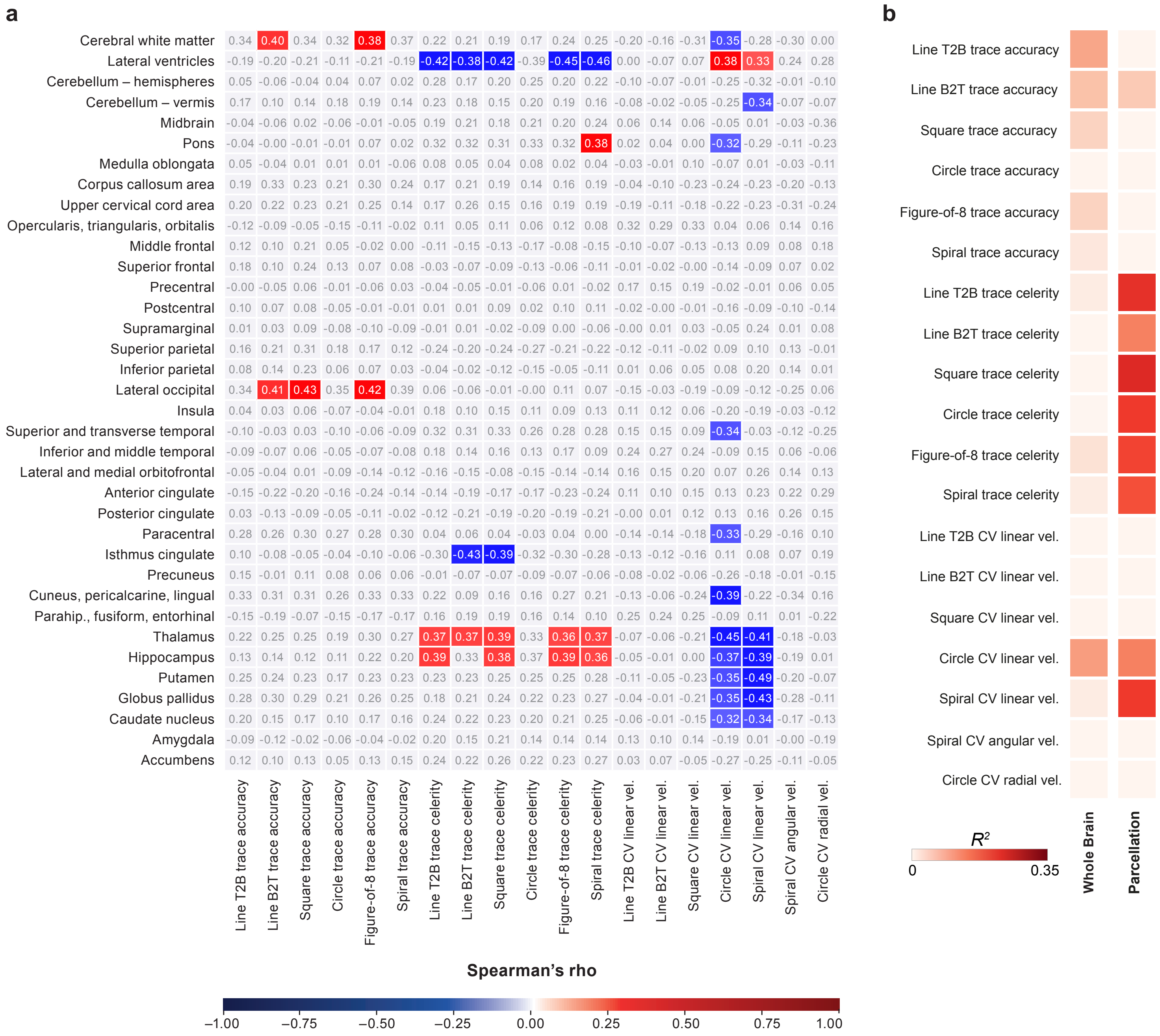

Fig. SI6 Spearman's rank correlation analysis and Bayesian ridge regression model for estimating the variance (R^2) of digital measures derived from the Draw a Shape Test

a Statistically significant (q<0.05) positive and negative correlations are highlighted in red and blue, respectively. FDR correction was applied for each digital and standard clinical measure separately to correct for multiple comparisons. Higher values equate to better performance for digital measures assessing trace accuracy and trace celerity. In contrast, higher values equate to worse performance for digital measures assessing CV linear, CV angular, and CV radial velocity. **b** Two Bayesian ridge regression models with leave-one-out cross validation were applied for estimating the variance (R^2) in the digital measures derived from the Draw a Shape Test that can be explained by volumetric MRI. The first model ("Whole Brain") included normalized brain volume and the three demographic variables: age, sex, and body mass index. The second model ("Parcellation") included all 36 regional MRI regions and the same three demographic variables. In particular, trace celerity and variability of linear velocity on round, complex shapes showed a larger R^2 score in the Parcellation model vs. in the Whole Brain model, which may reflect higher functional specificity.

B2T bottom to top, *CV* coefficient of variation, *FDR* false discovery rate, *parahip.* parahippocampus, *T2B* top to bottom, *vel.* drawing velocity

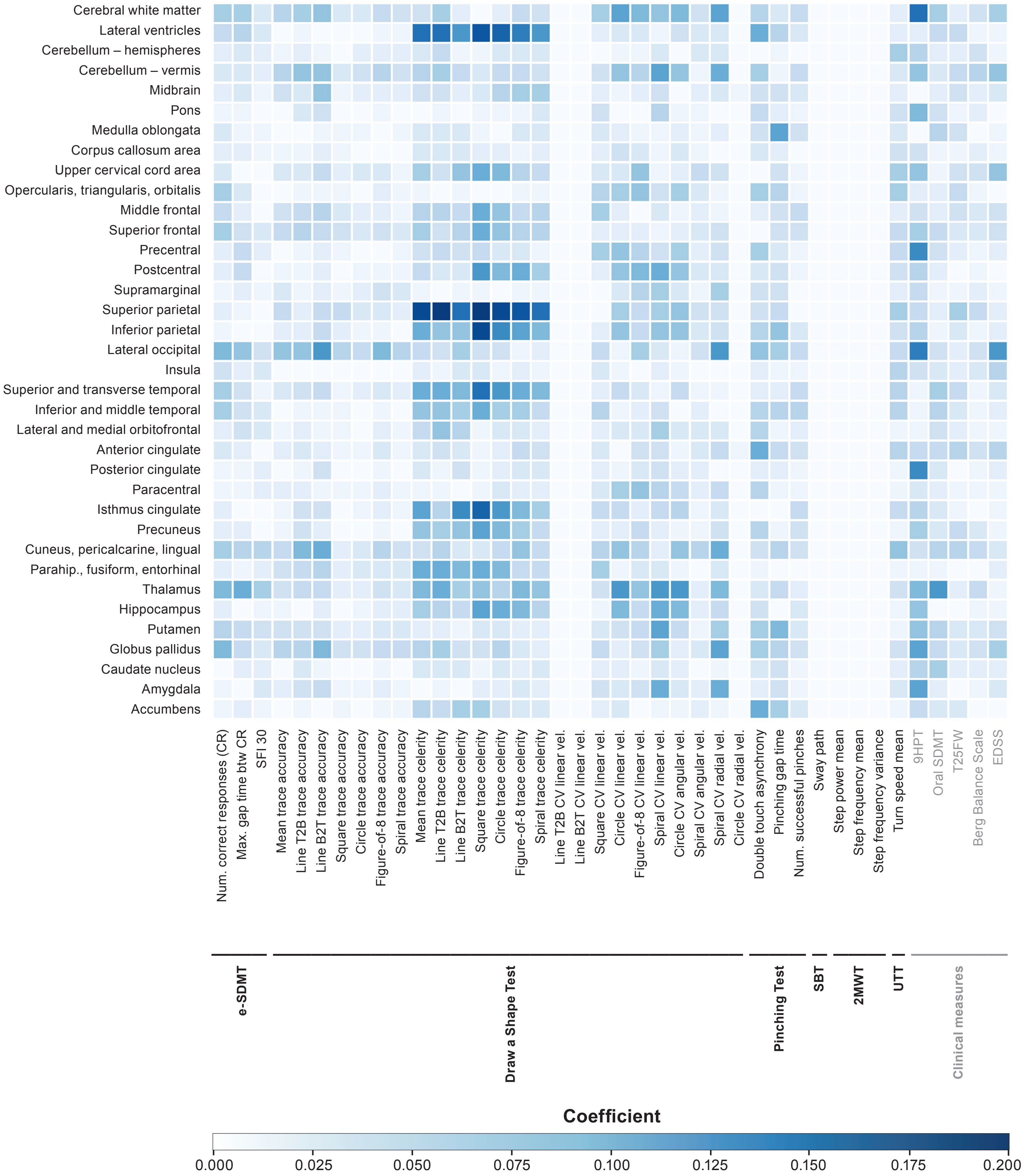

Fig. SI7 Bayesian ridge regression coefficients across the 36 regional MRI outcomes for digital measures and standard clinical measures

Higher values mean better performance on the oral SDMT and Berg Balance Scale, as well as for digital measures assessing number of correct responses and SFI 30 on the e-SDMT; trace accuracy and trace celerity on the Draw a Shape Test; number of pinches on the Pinching Test; mean step power and mean step frequency on the 2MWT; and mean turn speed on the UTT. In contrast, higher values equate to worse performance on the EDSS, 9HPT, and T25FW, as well as for digital measures assessing max. gap time between correct responses on the e-SDMT; CV linear, angular, and radial velocity on the Draw a Shape Test; double touch asynchrony and pinching gap time on the Pinching Test; sway path on the SBT; and step frequency variance on the 2MWT.

2MWT Two-Minute Walk Test, *9HPT* Nine-Hole Peg Test, *B2T* bottom to top, *btw* between, *CR* correct responses, *CV* coefficient of variation, *EDSS* Expanded Disability Status Scale, *e-SDMT* smartphone-based electronic Symbol Digit Modalities Test, max. maximum, *num*. number of, *parahip*. parahippocampus, *SBT* Static Balance Test, *SDMT* Symbol Digit Modalities Test, *SFI* speed fatigability index, *T2B* top to bottom, *T25FW* Timed 25-Foot Walk, *UTT* U-Turn Test, *vel*. drawing velocity

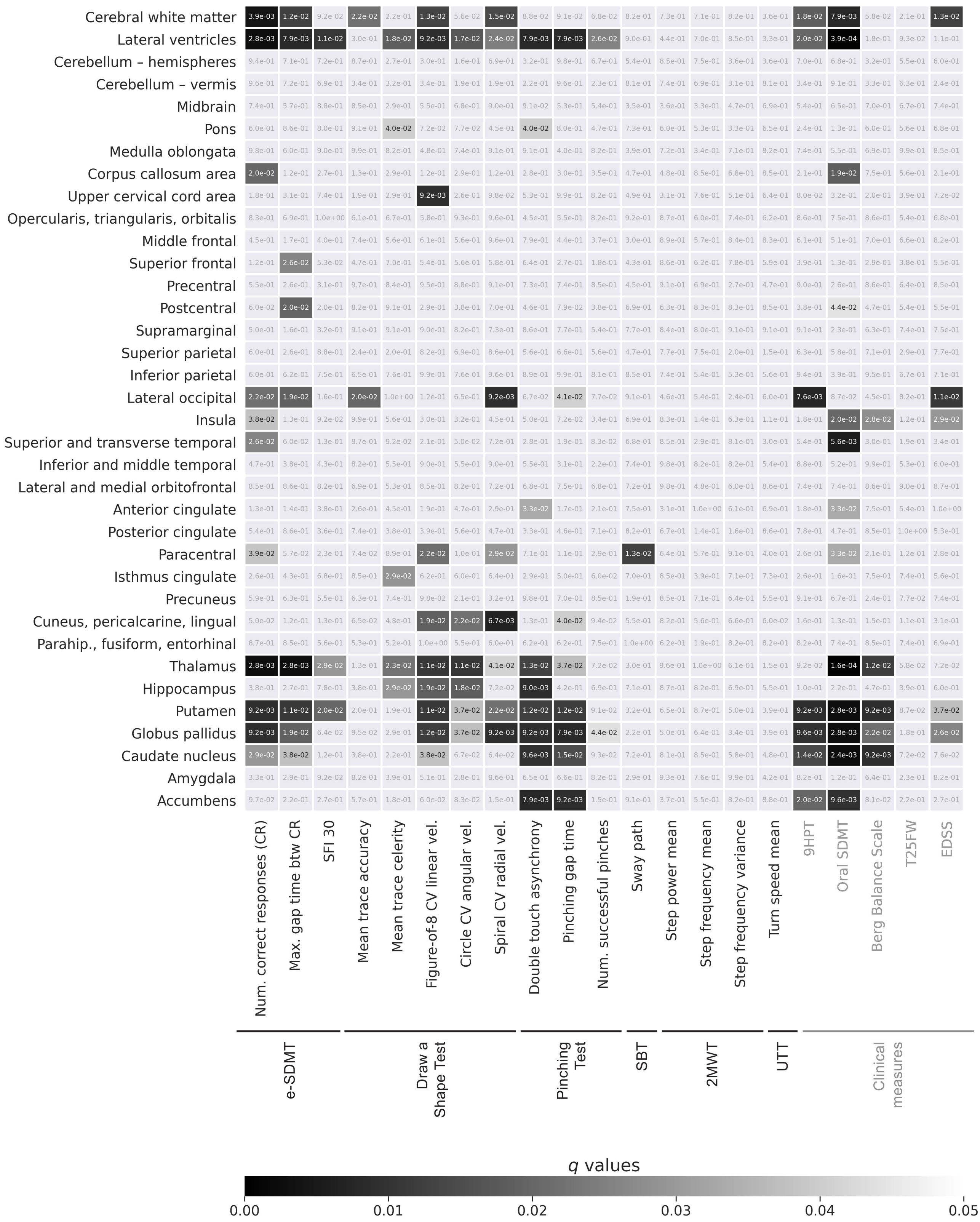

Fig. SI8 FDR-corrected *p* values (*q* values) associated with the Spearman's rank correlation analysis of digital measures and standard clinical measures with global and regional MRI outcomes

Statistical significance ($q<0.05$) is highlighted in grayscale. FDR correction is applied for all the possible combinations of the 36 anatomical regions and 21 digital/clinical measures (36 [MRI outcomes] × 21 [digital measures] configuration).

2MWT Two-Minute Walk Test, *9HPT* Nine-Hole Peg Test, *btw* between, *CR* correct responses, *CV* coefficient of variation, *EDSS* Expanded Disability Status Scale, *e-SDMT* smartphone-based electronic Symbol Digit Modalities Test, *FDR* false discovery rate, *max.* maximum, *num.* number of, *parahip.* parahippocampus, *SBT* Static Balance Test, *SDMT* Symbol Digit Modalities Test, *SFI* speed fatigability index, *T25FW* Timed 25-Foot Walk, *UTT* U-Turn Test, *vel.* drawing velocity
